# Cerebrovascular compromise and cognitive decline driven by chronic heart failure

**DOI:** 10.3389/fneur.2025.1644634

**Published:** 2025-10-28

**Authors:** Chenjia Zhao, Fengjun Du, Xiaoxia Hou, Guanhui Wu, Xiaoyu Cheng, Qingzhang Cheng, Hongxuan Feng, Hao Zhu, Meixia Wang

**Affiliations:** ^1^Department of Electrocardiogram, Gusu College of Nanjing Medical University, Affiliated Suzhou Hospital of Nanjing Medical University (Suzhou Municipal Hospital), Suzhou, China; ^2^Department of Epidemiology, School of Public Health, Qilu Hospital, Cheeloo College of Medicine, Shandong University, Jinan, Shandong, China; ^3^Department of Neurology, Gusu College of Nanjing Medical University, Affiliated Suzhou Hospital of Nanjing Medical University (Suzhou Municipal Hospital), Suzhou, China; ^4^Department of Neurology and Clinical Research Center of Neurological Disease, The Second Affiliated Hospital of Soochow University, Suzhou, China

**Keywords:** cognitive impairment, chronic heart failure, DTI, CSVD, heart failure

## Abstract

**Objective:**

This study investigated the effects of chronic heart failure with decreased LVEF on small cerebral vascular disease (CSVD) and associated cognitive impairment.

**Methods:**

This study encompassed a cohort of 73 patients diagnosed with chronic heart failure (CHF) at the Cardiovascular Department of Suzhou Municipal Hospital, spanning the period from June 2018 to August 2022. Participants were stratified into two groups based on a left ventricular ejection fraction (LVEF) threshold of 50%: 40 patients were categorized into the LVEF≥50% group, representing 54.8% of the cohort, while 33 patients were assigned to the LVEF<50% group, constituting 45.2% of the cohort. Each subject underwent a series of assessments, including brain magnetic resonance imaging (MRI), cardiac ultrasound, and neurocognitive function tests. For the analysis of diffusion tensor imaging (DTI), the tract-based spatial statistics (TBSS) method was employed to evaluate the DTI parameters of the participants’ brain white matter. Due to the extended duration of the DTI examination and the stringent requirements for patient compliance, only 37 patients, accounting for 50.7% of the total cohort, completed the DTI scans. This subset included 20 patients from the LVEF≥50% group and 17 patients from the LVEF<50% group.

**Results:**

In patients with congestive heart failure (CHF) and a left ventricular ejection fraction (LVEF) of less than 50%, the total Montreal Cognitive Assessment (MoCA) score was significantly lower compared to those with an LVEF of 50% or greater (*p* < 0.001). Among the four common types of cerebral small vessel disease (CSVD), the occurrence of white matter hyperintensities (WMH) (odds ratio = 0.228, 95% confidence interval [0.055, 0.949], *p* = 0.042) was found to be associated with LVEF in CHF patients, independent of the severity of WMH. Furthermore, LVEF in CHF patients demonstrated a positive correlation with total MoCA scores, as well as with visuospatial/executive functions, attention, and delayed recall. Tract-based spatial statistics (TBSS) analysis revealed that, in comparison to CHF patients with LVEF ≥ 50%, those with LVEF < 50% exhibited a significant decrease in fractional anisotropy (FA) and significant increases in mean diffusivity (MD), axial diffusivity (AD), and radial diffusivity (RD). In CHF patients, FA was positively correlated with visuospatial/executive functions, attention, and delayed recall, whereas MD, AD, and RD were negatively correlated with these cognitive domains.

**Conclusion:**

Chronic heart failure is significantly associated with the presence of CSVD (especially WMH) and the severity of cognitive impairment, with decreased LVEF correlating with worse outcomes.

## 1 Introduction

Chronic heart failure (CHF) is a syndrome characterized by a range of cardiac structural or functional disorders that result in subsequent systolic and/or diastolic dysfunction, leading to inadequate perfusion of organs and tissues (1). In this study, patients with heart failure (HF) were classified based on left ventricular ejection fraction (LVEF) into those with preserved ejection fraction (HFpEF; LVEF ≥ 50%) and those with reduced ejection fraction (LVEF < 50%), with the latter serving as a critical threshold for assessing cardiac function impairment (2).

A reduction in cardiac output disrupts cerebral blood flow (CBF) homeostasis (3). Hooghiemstra et al. introduced the concept of the “heart-brain axis,” positing that the hemodynamic equilibrium within this axis is crucial for maintaining the structural and functional integrity of the brain (4). Hemodynamic dysfunction or abnormalities within any component of the “heart-brain axis” may constitute a risk factor for vascular brain injury, potentially leading to the development of cerebrovascular diseases such as cerebral small vessel disease (CSVD), stroke, vascular cognitive impairment (VCI), or even vascular dementia (VaD) (5). Some studies even suggest that heart failure is an independent risk factor for cognitive impairment, and chronic hypoperfusion has been considered as a potential pathophysiological mechanism (6, 7). However, there are few studies about the effect of heart failure on the magnetic resonance imaging (MRI) manifestations of CSVD, including white matter hyperintensities (WMH), even have shown conflicting results (8).

Cerebral small vessel diseases (CSVD) refer to a series of clinical, imaging, and pathological syndromes characterized by damage to the white matter and deep gray matter of the brain caused by intracranial small vessel lesions, and is mainly diagnosed by neuroimaging techniques, such as magnetic resonance imaging (MRI) (9). CSVD results in characteristic MRI features, including cerebral microbleeds (CMB), white matter hyperintensities (WMH), lacunar infarcts (LI), enlarged perivascular spaces (EPVS), and brain atrophy (10). It has been demonstrated that diffusion tensor imaging (DTI) is particularly sensitive in detecting white matter damage in CSVD both within T2- WMH and in apparently “normal appearing white matter” (11). At present, the evaluation of complex structure networks through Functional magnetic resonance imaging (MRI) detection is mainly based on graph theory analysis, and network information is quantified by DTI parameters (12). Based on the anisotropy of water molecules’ diffusion motion, DTI quantifies the diffusion measurement and direction of water in three-dimensional space through main parameters such as fractional anisotropy (FA), mean diffusivity (MD), radial diffusivity (RD) and axial diffusivity (AD), thereby reflecting the structural and functional changes of brain white matter (13).

Based on the controversial results of studies exploring the association between cardiac function changes and MRI manifestations of CSVD (8), we attempted to describe the structural and functional variations of white matter in chronic heart failure patients complicated by CSVD through DTI detection. We hope to provide theoretical support for studying the pathophysiological mechanism of chronic heart failure complicated with CSVD in-depth.

## 2 Materials and methods

### 2.1 Subjects and data collection

This is an observational and prospective study that included randomly recruited patients with clinically diagnosed chronic HF at the Cardiovascular and Neurology Clinics of Suzhou Municipal Hospital from June 2018 to August 2022. The inclusion criteria are shown as follows: (a) 40 years old and above, no upper age limit; (b) clinical diagnosis of chronic HF in the New York Heart Association (NYHA) functional class II–IV; (c) ability to undergo MRI scanning; (d) informed Consent available. The exclusion criteria: (a) Left ventricle ejection fraction≤35%; (b) Atrial fibrillation or other indication for long-term anticoagulation; (c) Severe anemia, coagulopathies or chronic anticoagulation; (d) Valvular heart disease; (e) psychiatric illness or alcoholism; (f) thyroid dysfunction, liver cirrhosis, hepatic encephalopathy and other diseases affecting cognitive function; (g) Platelets <100 (109/L); (h) Any contraindication to MRI detecting.

General data collection includes gender, age, vascular risk factors (such as hypertension, dyslipidaemia, diabetes and smoking). Laboratory data included fasting blood glucose (mmol/L), urea (mmol/L), creatinine (umol/L), triglyceride (mmol/L), total cholesterol (mmol/L), low-density lipoprotein cholesterol (LDL-C, mmol/L) and B-type natriuretic peptide (BNP, pg./ml). Medical treatment includes whether to take aspirin, clopidogrel, statins, angiotensin-converting enzyme inhibitor (ACEI), angiotensin receptor blocker (ARB), calcium channel blocker (CCB) and Beta-blockers. The patient underwent multiple tests, including neurocognitive assessment, Doppler echocardiographic examination and MRI. The experimental subjects were divided according to previously established cut-off value of LVEF (50%) (2).

**Figure 1:**
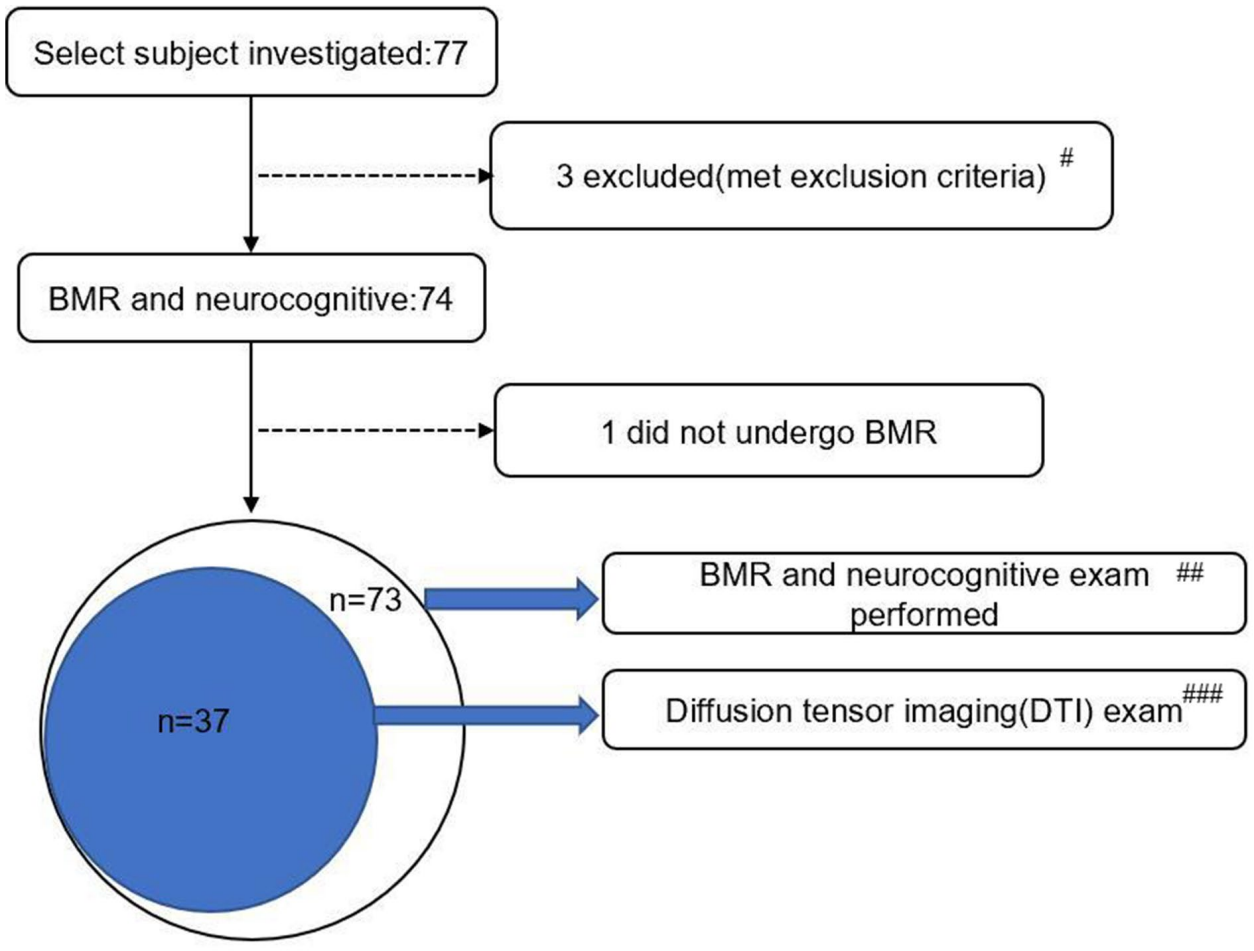
Study flowchart. BMR, brain magnetic resonance; DTI, diffusion tensor imaging. #Exclusion reasons: 1 case with LVEF ≤35%, 1 case with atrial fibrillation requiring long-term anticoagulation, 1 case with severe anemia. ##Subgroup sample size: EF ≥ 50% group (*n* = 40), EF < 50% group (*n* = 33). ###DTI subgroup distribution: EF ≥ 50% group (*n* = 20), EF < 50% group (*n* = 17).

A total of 77 patients with CHF were initially included in the study (Figure 1). Among them, 3 individuals who met the exclusion criteria were excluded (1 with LVEF ≤35%, 1 with atrial fibrillation requiring long-term anticoagulation, and 1 with severe anemia), leaving 74 individuals for further testing. Ultimately, 73 patients (EF ≥ 50% group: *n* = 40; EF < 50% group: *n* = 33) completed brain MRI and neurocognitive assessment, with 1 patient unable to undergo brain MRI due to claustrophobia.

Due to the extended duration of the Diffusion Tensor Imaging (DTI) examination, which lasted 9 min and 29 s, and the high level of participant cooperation required, approximately 50% of participants were randomly selected from two groups, namely those with an ejection fraction (EF) of 50% or greater and those with an EF of less than 50%, to undergo further DTI examination. As a result, 37 patients completed the DTI examinations, comprising 20 from the EF ≥ 50% group and 17 from the EF < 50% group. The selection of the DTI subgroup was conducted using stratified random sampling, based on the grouping ratio of the entire cohort (EF ≥ 50%: EF < 50% ≈ 1.2:1). Specifically, random numbers were generated for patients within each left ventricular ejection fraction (LVEF) group using the random number table function in Excel. The top 50% of patients with valid random numbers were selected to complete the DTI scans, ensuring that the subgroup’s grouping ratio was consistent with that of the total cohort (EF ≥ 50%: EF < 50% = 20:17 ≈ 1.18:1). The baseline characteristics of the DTI subgroup were balanced between the two groups (all *p* > 0.05), thereby ensuring the comparability of the DTI parameter results. No significant differences were observed between the EF ≥ 50% and EF < 50% groups in the DTI subgroup for age (74.5 ± 4.0 vs. 74.1 ± 3.8 years, t = 0.36, *p* = 0.782), gender (male ratio: 55.0% vs. 58.8%, χ^2^ = 0.08, *p* = 0.801), vascular risk factors (hypertension: 60.0% vs. 64.7%, *p* = 0.812; diabetes: 35.0% vs. 41.2%, *p* = 0.765), MoCA total score (24.3 ± 2.1 vs. 21.9 ± 2.7, *p* < 0.001, consistent with the total cohort), and laboratory indicators (BNP: 632.5 ± 428.3 vs. 678.2 ± 445.1 pg./mL, *p* = 0.736). These results confirm that the two groups in the DTI subgroup maintained the same baseline balance as the total cohort (Table 1), ensuring comparability of DTI parameters. To rule out selection bias, we further compared baseline characteristics between the DTI subgroup (*n* = 37) and the 36 patients who did not complete DTI scans (non-DTI group). No significant differences were found in age (74.3 ± 3.9 vs. 74.4 ± 4.1 years, *p* = 0.921), gender (male ratio: 56.8% vs. 55.6%, *p* = 0.938), vascular risk factors (all *p* > 0.05), MoCA total score (23.2 ± 2.6 vs. 23.1 ± 3.0, *p* = 0.897), or LVEF distribution (EF ≥ 50%: EF < 50% = 20:17 vs. 20:16, *p* = 0.982) between the two groups. This confirms that the DTI subgroup is representative of the total cohort, and the exclusion of non-DTI patients did not introduce selection bias.

**Table 1:** Baseline characteristics of the population.

Variables	All (*n* = 73)	LVEF≥50% (*n* = 40)	LVEF<50% (*n* = 33)	*p*-value
Clinical characteristics				
Age	74.32 ± 3.94	74.60 ± 4.18	73.97 ± 3.67	0.501
Male sex (*n* (%))	41 (56.16%)	22 (55.00%)	19 (57.58%)	0.828
Vascular risk factors				
Hypertension (*n* (%))	40 (67.12%)	25 (62.50%)	22 (66.67%)	0.716
Dyslipidaemia (n (%))	43 (58.90%)	24 (60.00%)	19 (57.57%)	0.837
Diabetes (*n* (%))	29 (39.73%)	14 (35.00%)	15 (45.45%)	0.371
Smoking (*n* (%))	41 (56.16%)	23 (57.50%)	18 (54.55%)	0.803
Laboratory studies				
Fasting blood glucose (mmol/L)	5.96 ± 1.28	5.83 ± 1.23	6. 11 ± 1.34	0.357
Urea (mmol/L)	7.22 ± 4.09	7.08 ± 4.23	7.38 ± 3.98	0.759
Creatinine (umol/L)	74.38 ± 23.60	73.52 ± 23.47	75.42 ± 24.08	0.734
Triglyceride (mmol/L)	1.37 ± 0.95	1.39 ± 1.12	1.34 ± 0.71	0.810
Total cholesterol (mmol/L)	4. 12 ± 1.05	4.06 ± 1.19	4.19 ± 0.85	0.602
LDL-C (mmol/L)	2.65 ± 0.94	2.54 ± 1.02	2.79 ± 0.83	0.266
BNP (pg/mL)	654.63 ± 438.05	638.33 ± 432.16	674.39 ± 451.01	0.729
Medical treatment				
Aspirin (*n* (%))	51 (69.86%)	28 (70.00%)	23 (69.70%)	0.978
Clopidogrel (*n* (%))	20 (27.40%)	12 (30.00%)	8 (24.24%)	0.589
Statins (*n* (%))	56 (76.71%)	31 (77.50%)	25 (75.76%)	0.863
ACEI (*n* (%))	21 (28.76%)	12 (30.00%)	9 (27.27%)	0.801
ARB (*n* (%))	27 (36.99%)	14 (35.00%)	13 (39.39%)	0.704
CCB (*n* (%))	20 (27.40%)	12 (30.00%)	8 (24.24%)	0.589
β-blockers (*n* (%))	42 (57.53%)	24 (60.00%)	18 (54.55%)	0.644
MoCA score	23.22 ± 2.85	24.40 ± 2.23	21.79 ± 2.89	<0.001

All data are shown as *n* (%) or mean (±standard deviation). BNP, B type natriuretic peptide, LDL-C, low-density lipoprotein cholesterol; HDL, the high density lipoprotein; NYHA, the New York Heart Association; LVEF, the left ventricular ejection fraction; ACEI, angiotension-converting enzyme inhibitor; ARB, angiotension receptor bloker; CCB, calcium channel blocker; MoCA, the Montreal Cognitive Assessment.

The Ethics Committee of Affiliated Suzhou Hospital of Nanjing Medical University (Suzhou Municipal Hospital) approved this study (Ethics Review Number: K-2021-029). All subjects were right-handed. Each person accepted and signed an informed consent form before the study.

### 2.2 Neurocognitive assessment

The Montreal Cognitive Assessment (MoCA) is a rapid screening tool for assessing cognitive dysfunction (14). It evaluates different cognitive domains, such as visuospatial/ executive functions (5 points), attention (6 points), abstraction (2 points), naming (3 points), language (3 points), orientation (6 points) and delayed recall (5 points). Add up the scores of each item to obtain the total score. If the patient has been educated less than 12 years, the total score will be added by 1 point. A total score ≥26 is considered normal. The maximum total score is 30 points. A higher score indicates better cognitive function (15).

### 2.3 Image acquisition

Neuroimaging data were all acquired on a 3.0-Tesla MRI scanner equipped with an 8-channels head coil (Achieva TX, Philips Healthcare, Best, the Netherlands). The participants were performed in the supine position with their heads fixed, their eyes closed, and they remained awake during the scan. The MRI images were screened rapidly by professional imaging diagnostic physicians, and cases with obvious intracranial organic lesions (2 cases were found in this study) were preliminarily excluded. In addition, all subjects underwent T1- weighted, susceptibility weighted imaging (SWI), fluid-attenuated inversion recovery (FLAIR) and DTI to acquire imaging data. All image acquisitions were performed on the same MR scanner and scanned by trained stationary technicians. The identification of all four MRI markers (Lacunes, WMHs, PVSs, and CMBs) for CSVD referred to previously reported neuroimaging standards (16).

Imaging judgment methods for cerebral microbleeds (17): ① Appearing as small circular or elliptical shapes on SWI, with clear boundaries, homogeneity, and no obvious peripheral tissue edema, generally limited to short low signals on one or two consecutive layers, with a diameter range of 2–5 mm; ② Mainly distributed in the basal ganglia area, cerebral cortex, thalamus, brainstem, cerebellum, etc., and can occur simultaneously in multiple parts; ③ Exclude other similar artifacts, such as vascular flow voids, ferroflavin deposits, or small calcifications. The Fazekas scoring scale is recommended for clinical evaluation of white matter hyperintensities (44). This scale is based on head MRI-T2WI or FLAIR sequence images, scoring paraventricular and deep white matter lesions separately (0–3 points each), with total scores ranging from 0 to 6 points. Higher scores indicate more severe WMH, classified as mild (0–1 points), moderate (3–4 points), and severe (5–6 points).

Lacunar infarcts appears as circular or ovoid 3–15 mm lacunar like lesions on MRI, surrounded by high signal circular halos. The lesions show low signal on MRI-T1 and FLAIR sequences, and high signal on T2 sequences. The MRI manifestation of enlarged perivascular spaces (EPVS) is a well-defined circular or linear space that is consistent with the shape of the arterioles, with a diameter of less than 3 mm. PVS shows low signal on T1Wl and FLAIR, and high signal on T2Wl, similar to the cerebrospinal fluid signal. The MRI sequence parameters are described as follows (Table 2).

**Table 2:** Description of BMR sequence characteristics.

MRI sequence	Sequence parameters
Axial 3D magnetization-prepared rapid gradient-echo imaging (MPRAGE) MRI sequence T1-weighted	TR = 2,300 ms, TE = 2.98 ms, TI = 900 ms, flip angle = 9°, FOV = 256×256 mm, matrix = 122×122, NEX = 1, number of slices = 176, slice thickness = 1.10 mm, scanning time = 5:12
Sagittal 3D fluid-attenuated inversion recovery (FLAIR) sequence	TR = 8,000 ms, TE = 81 ms, TI = 2,370 ms, flip angle = 150°, FOV = 220x220mm, matrix = 122×122, NEX = 1, number of slices = 20, slice thickness = 5 mm, scanning time = 2:26
Axial 3D susceptibility-weighted (SWI) sequence	TR = 77 ms, TE = 49 ms, flip angle = 20°, FOV = 22×22 cm, matrix = 256×256, 0.7 NEX, number of slices = 108, slice thickness = 3 mm, scanning time = 6:27
Axial diffusion tensor (DTI) echo-planar spin-echo sequence	TR = 5,400 ms, TE = 93 ms, flip angle = 90°, FOV = 220mmx220mm, matrix = 122×122, NEX = 1, number of slices = 40, slice thickness = 4 mm, b = 1,000 s/mm^2^, number of diffusion directions = 30, scanning time = 9:29

FA, flip angle; TR, repetition time; TE, time to echo; TI, inversion time; FOV, field of view; NEX, number of excitations.

### 2.4 Data pre-processing

The DTI data were preprocessed by FSL software (18). The steps are as follows: First, convert the original DICOM data into NIFTI format using dcm2nii; Next, eliminate head movement and deformation caused by eddy currents, and use linear registration and alignment for B0 images; Then, adjust the original gradient direction based on the changes in eddy current correction; Obtain brain masks to remove non-brain tissue, improving spatial alignment accuracy, limiting the analysis range and reducing the amount of operations; Last, calculate the tensor by using the dtifit function in FSL and obtain the relevant metrics (FA, MD, AD, and RD).

### 2.5 Tract-based spatial statistics

By FSL software, individual FAs were aligned to the standard space using linear and nonlinear alignments (19). Next, based on the FA aligned with the standard space, the average FA map and white matter skeleton were constructed. Align the white matter fibers of each subject with the average FA template fiber skeleton image, then extract the FA fiber skeleton image and superimpose it onto the corresponding structural image, and convert the spatial position coordinates into MNI space for automatic anatomical localization. Average diffusion metrics (FA, MD, AD and RD) were extracted from each subject’s white matter skeleton. Two sample *t*-tests were performed using the Glm tool. Conduct statistics using a permutation test of 5,000 permutations. Multiple comparisons were corrected through the TFCE method, and 0.05 was used as the significance threshold for the modified *p*-value. Finally, JHU White Matter Tractography Atlas was used to identify the clumps with significant differences, and the results were visualized using the inflated clumps.

### 2.6 Statistical analysis

All data were analyzed by IBM SPSS statistics (version 22). For continuous variables, satisfying a normal distribution, the mean ± standard deviation was used. The median (interquartile spacing) was used if the normal distribution was not satisfied, and for categorical variables, *n* (%) was used. For comparative analysis between groups, independent sample *t*-test was used for continuous variables satisfying normal distribution and chi-square test was used for categorical variables. The correlation analysis between Rich club network parameters and MoCA scores of each group was conducted by Pearson after controlling gender and age. When *p* < 0.05, the difference was statistically significant. For multiple comparisons in TBSS analysis, a permutation test with 5,000 permutations and TFCE (Threshold-Free Cluster Enhancement) correction was used (*p* < 0.05). For multiple CSVD subtype analyses (CMB, LI, WMH, EPVS), Bonferroni correction was applied to adjust the significance threshold to *p* < 0.0125 (0.05/4) to reduce false-positive results.

## 3 Results

### 3.1 Baseline characteristics of the participants

Baseline clinical characteristics, comorbidities, blood tests, and medical histories were assessed for each participant. The experimental cohort was stratified into two groups based on left ventricular ejection fraction (LVEF) at a threshold of 50%. No significant differences were found between the two groups concerning age, gender, vascular risk factors (including hypertension, dyslipidemia, diabetes, and smoking), laboratory test results, or treatment history (*p* > 0.05). The Montreal Cognitive Assessment (MoCA) scores for heart failure (HF) patients in both groups were below the normal range (<26 points). A comparison of MoCA scores between the two groups revealed that the group with LVEF <50% had significantly lower scores than the group with LVEF ≥50% (*p* < 0.001). Detailed results are presented in Table 1. All participants were right-handed. Over a six-month follow-up period, no clinical events (such as myocardial infarction, stroke, percutaneous coronary intervention, or death) were reported among the subjects.

### 3.2 Correlation between CSVD and LVEF in CHF patients

In this study, we examined two cohorts of CHF patients using four distinct MRI imaging modalities for assessing CSVD, as detailed in Table 3a. Our analysis revealed no significant difference in the prevalence of EPVDS complications between the two groups (*p* > 0.05). However, significant differences were observed in the prevalence of CMB, LI, and WMH (*p* < 0.05). We further explored the relationship between various CSVD imaging types and CHF. A multivariable logistic regression model was constructed, controlling for age, gender, hypertension, diabetes mellitus, dyslipidemia, and smoking as covariates, with the results presented in Table 3b. The analysis demonstrated that WMH incidence (OR = 0.228, 95% CI [0.055, 0.949], *p* = 0.042) remained significantly associated with the LVEF in CHF patients. Using Fazekas scores, WMH severity was classified into normal, mild, moderate, or severe categories. Subsequently, CHF subjects were stratified based on WMH severity, as shown in Table 3c, and the comparison revealed no significant statistical differences between the groups.

**Table 3:** (a) Differences in imaging types of CSVD in CHF, (b) The relationship between different imaging types of CSVD and LVEF, and (c) Difference of different severity of WMH in CHF.

(a)				
Imaging types of CSVD	All (*n* = 73)	LVEF≥50% (*n* = 40)	LVEF<50% (*n* = 33)	*p*-value
CMBs (*n* (%))	32 (43.84%)	13 (32.50%)	19 (57.58%)	0.032
LI (*n* (%))	22 (30. 14%)	8 (20.00%)	14 (42.42%)	0.038
WMH (*n* (%))	44 (60.27%)	18 (45.00%)	26 (78.78%)	0.003
EPVS (*n* (%))	30 (41. 10%)	14 (35.00%)	16 (48.48%)	0.250

(b)		
Imaging types of CSVD	OR (95%CI)	*p*-value
CMBs (*n*)	2.815 (1.003, 7.902)	0.049
LI (*n*)	3.1817 (1.089, 9.326)	0.035
WMH (*n*)	4.392 (1.053, 18.286)	0.042

(c)				
Imaging types of CSVD	All (*n* = 73)	LVEF≥50% (*n* = 40)	LVEF<50% (*n* = 33)	*p*-value
Normal (*n* (%))	29 (39.73%)	22 (55.00%)	7 (21.21%)	–
Mild (*n* (%))	15 (20.55%)	7 (17.50%)	8 (24.24%)	0.485
Moderate (*n* (%))	15 (20.55%)	6 (15.00%)	9 (27.27%)	0.202
Severe (*n* (%))	14 (19. 18%)	5 (12.50%)	9 (27.27%)	0.114

All data are shown as *n* (%). CMBs, Cerebral Microbleeds; LI, Lacunar Infarction; WMH, White Matter Hyperintensities; EPVS, Enlarged Perivascular Space.

### 3.3 Correlation between cognitive scale scores and LVEF in CHF patients

Correlation analyses between the scores of MoCA scale’s each item and LVEF were conducted. The results showed that LVEF in CHF patients was positively correlated with total scores of MoCA, visuospatial/ executive functions, attention and delayed recall (Table 4).

**Table 4:** The relationship between the scores of MoCA scale’s each item and LVEF.

Item	MoCA	Visuospatial/executive functions	Attention	Naming	Abstraction	Language	Delayed recall	Orientation
*r*	0.453***	0.269*	0.278*	0.157	0.095	0.024	0.320**	0.113
*p*-value	<0.001	0.021	0.017	0.186	0.424	0.838	0.006	0.339

LVEF, the left ventricular ejection fraction; MoCA, the Montreal Cognitive Assessment. *** *p* < 0.001, ** *p* < 0.01, * *p* < 0.05.

### 3.4 Comparison of FA, MD, AD, and RD

**Figure 2:**
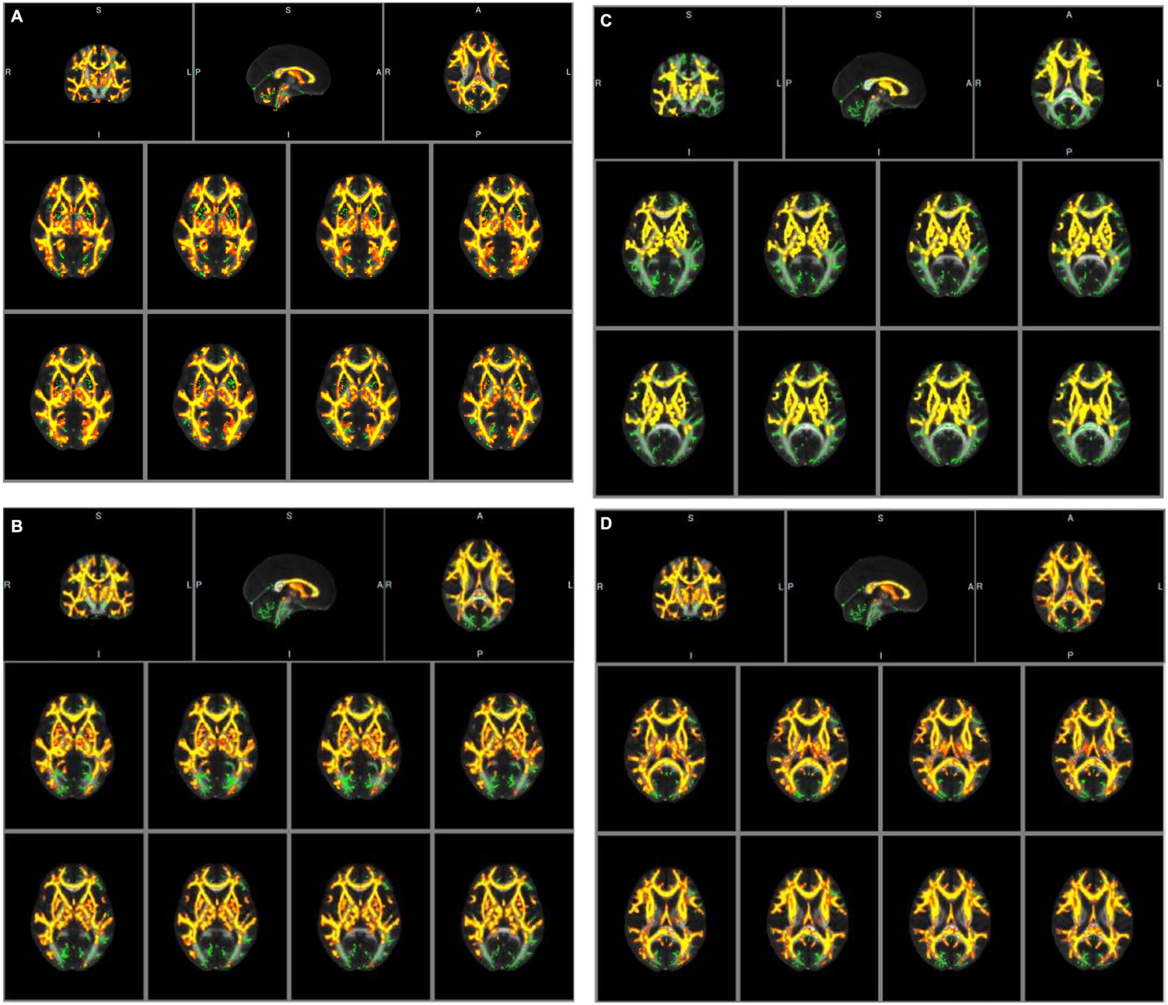
**(A)** Tract-based spatial statistic results of fractional anisotropy (FA) images between the two groups of CHF patients. Green represents mean skeleton of all participants; red and yellow represents regions with decreased FA in CHF patients with LVEF<50% (*p* < 0.05, TFCE corrected for multiple comparisons). **(B)** Tract-based spatial statistic results of mean diffusivity (MD) images between the two groups of CHF patients. Green represents mean skeleton of all participants; red and yellow represents regions with increased MD in CHF patients with LVEF<50% (*p* < 0.05, TFCE corrected for multiple comparisons). **(C)** Tract-based spatial statistic results of axial diffusivity (AD) images between the two groups of CHF patients. Green represents mean skeleton of all participants; red and yellow represents regions with increased AD in CHF patients with LVEF<50% (*p* < 0.05, TFCE corrected for multiple comparisons). **(D)** Tract-based spatial statistic results of radial diffusivity (RD) images between the two groups of CHF patients. Green represents mean skeleton of all participants; red and yellow represents regions with increased RD in CHF patients with LVEF<50% (*p* < 0.05, TFCE corrected for multiple comparisons).

An analysis of baseline data, including age, gender, vascular risk factors, laboratory testing data, and treatment history, for participants undergoing diffusion tensor imaging (DTI) examination revealed no statistically significant differences (*p* > 0.05). However, tract-based spatial statistics (TBSS) analysis identified significant differences in all diffusion indicators between the groups. Specifically, in patients with congestive heart failure (CHF) and left ventricular ejection fraction (LVEF) < 50%, fractional anisotropy (FA) decreased significantly, whereas mean diffusivity (MD), axial diffusivity (AD), and radial diffusivity (RD) increased significantly, compared to CHF patients with LVEF≥50% (see Tables 5a–d; Figures 2A–D).

**Table 5:** (a) Difference of fractional anisotropy (FA), (b) Difference of mean diffusivity (MD), (c) Difference of axial diffusivity (AD), and (d) Difference of radial diffusivity (RD).

Cluster Index	Voxels/mm^3^	MNI			*p*-value
		X (vox)	Y (vox)	Z (vox)	
(a)					
1	90,030	11	31	−15	
JHU White-Matter Tractography Atlas		Average probability			
Anterior thalamic radiation L		0.995824			<0.001
Anterior thalamic radiation R		1.03925			<0.001
Corticospinal tract L		0.484261			<0.001
Corticospinal tract R		0.446762			<0.001
Cingulum (cingulate gyrus) L		0.279996			<0.001
Cingulum (cingulate gyrus) R		0.157847			<0.001
Cingulum (hippocampus) L		0.080662			<0.001
Cingulum (hippocampus) R		0.113762			<0.001
Forceps major		0.887593			<0.001
Forceps minor		2.56693			<0.001
Inferior fronto-occipital fasciculus L		1.34713			<0.001
Inferior fronto-occipital fasciculus R		1.58891			<0.001
Inferior longitudinal fasciculus L		0.959458			<0.001
Inferior longitudinal fasciculus R		0.830023			<0.001
Superior longitudinal fasciculus L		1.28377			<0.001
Superior longitudinal fasciculus R		1.29589			<0.001
Uncinate fasciculus L		0.517639			<0.001
Uncinate fasciculus R		0.257692			<0.001
Superior longitudinal fasciculus (temporal part) L		0.592869			<0.001
Superior longitudinal fasciculus (temporal part) R		0.518672			<0.001
(b)					
1	74,215	10	55	−16	
JHU White-Matter Tractography Atlas		Average probability			
Anterior thalamic radiation L		1.35295			<0.001
Anterior thalamic radiation R		1.26175			<0.001
Corticospinal tract L		0.584828			<0.001
Corticospinal tract R		0.471158			<0.001
Cingulum (cingulate gyrus) L		0.167688			<0.001
Cingulum (cingulate gyrus) R		0.147544			<0.001
Cingulum (hippocampus) L		0.0339554			<0.001
Cingulum (hippocampus) R		0.10514			<0.001
Forceps major		0.280536			<0.001
Forceps minor		2.92762			<0.001
Inferior fronto-occipital fasciculus L		1.22811			<0.001
Inferior fronto-occipital fasciculus R		1.47396			<0.001
Inferior longitudinal fasciculus L		0.886249			<0.001
Inferior longitudinal fasciculus R		0.656597			<0.001
Superior longitudinal fasciculus L		1.91494			<0.001
Superior longitudinal fasciculus R		1.64088			<0.001
Uncinate fasciculus L		0.610227			<0.001
Uncinate fasciculus R		0.304211			<0.001
Superior longitudinal fasciculus (temporal part) L		0.800323			<0.001
Superior longitudinal fasciculus (temporal part) R		0.622866			<0.001
(c)					
1	38,475	23	−9	13	
JHU White-Matter Tractography Atlas		Average probability			
Anterior thalamic radiation L		1.98079			0.004
Anterior thalamic radiation R		1.85149			0.004
Corticospinal tract L		0.820299			0.004
Corticospinal tract R		0.573229			0.004
Cingulum (cingulate gyrus) L		0.132658			0.004
Cingulum (cingulate gyrus) R		0.0184795			0.004
Cingulum (hippocampus) L		0.0117355			0.004
Cingulum (hippocampus) R		0.0933593			0.004
Forceps major		0.00792723			0.004
Forceps minor		2.8033			0.004
Inferior fronto-occipital fasciculus L		0.894711			0.004
Inferior fronto-occipital fasciculus R		1.38737			0.004
Inferior longitudinal fasciculus L		0.0866017			0.004
Inferior longitudinal fasciculus R		0.608239			0.004
Superior longitudinal fasciculus L		2.53198			0.004
Superior longitudinal fasciculus R		1.91917			0.004
Uncinate fasciculus L		0.57245			0.004
Uncinate fasciculus R		0.348694			0.004
Superior longitudinal fasciculus (temporal part) L		0.952982			0.004
Superior longitudinal fasciculus (temporal part) R		0.651722			0.004
(d)					
1	84,710	17	34	−12	
JHU White-Matter Tractography Atlas		Average probability			
Anterior thalamic radiation L		1.19358			<0.001
Anterior thalamic radiation R		1.15899			<0.001
Corticospinal tract L		0.490703			<0.001
Corticospinal tract R		0.473241			<0.001
Cingulum (cingulate gyrus) L		0.214826			<0.001
Cingulum (cingulate gyrus) R		0.16537			<0.001
Cingulum (hippocampus) L		0.0642495			<0.001
Cingulum (hippocampus) R		0.117838			<0.001
Forceps major		0.673357			<0.001
Forceps minor		2.70512			<0.001
Inferior fronto-occipital fasciculus L		1.3532			<0.001
Inferior fronto-occipital fasciculus R		1.6212			<0.001
Inferior longitudinal fasciculus L		0.982928			<0.001
Inferior longitudinal fasciculus R		0.793426			<0.001
Superior longitudinal fasciculus L		1.70732			<0.001
Superior longitudinal fasciculus R		1.51486			<0.001
Uncinate fasciculus L		0.563134			<0.001
Uncinate fasciculus R		0.29708			<0.001
Superior longitudinal fasciculus (temporal part) L		0.741018			<0.001
Superior longitudinal fasciculus (temporal part) R		0.583535			<0.001

1. This table is based on the DTI subgroup (total *n* = 37), including 20 patients in the EF≥50% group and 17 patients in the EF<50% group. 2. The DTI subgroup was randomly selected from the total CHF sample (*n* = 73), with no significant differences in baseline characteristics (age, gender, vascular risk factors, laboratory indicators, medication use) between the two groups (all *p* > 0.05). 3. The DTI examination duration was 9 min and 29 s; some patients failed to complete the examination due to high cooperation requirements.

### 3.5 Correlation between diffusion indicators and MoCA scale in CHF patients

Correlation analysis demonstrated that all diffusion indicators were associated with the total score of the Montreal Cognitive Assessment (MoCA). FA exhibited a positive correlation with the MoCA score, while MD, AD, and RD showed negative correlations. Among the various components of the MoCA score, FA was positively correlated with visuospatial/executive functions, attention, and delayed recall, whereas MD, AD, and RD were negatively correlated with these cognitive domains (refer to Tables 6a,b).

**Table 6:** (a) The relationship between diffusion indicators and MoCA score (total score and each item) in CHF (*r*-value) and (b) The relationship between diffusion indicators and MoCA score (total score and each item) in CHF (*p*-value).

(a)								
*r*-value	MoCA	Visuospatial/executive functions	Attention	Naming	Abstraction	Language	Delayed recall	Orientation
FA	0.588	0.599	0.501	0.436	0.062	0.197	0.482	0.252
MD	−0.487	−0.502	−0.381	−0.285	−0.080	−0.070	−0.410	−0.262
AD	−0.471	−0.488	−0.349	−0.104	−0.099	−0.034	−0.401	−0.280
RD	−0.500	−0.515	−0.401	−0.208	−0.074	−0.097	−0.420	−0.251

(b)								
*p-*value	MoCA	Visuospatial/executive functions	Attention	Naming	Abstraction	Language	Delayed recall	Orientation
FA	<0.001^***^	<0.001^***^	0.002^**^	0.491	0.718	0.244	0.003^**^	0.133
MD	0.002^**^	0.002^**^	0.020^*^	0.322	0.636	0.679	0.012^*^	0.117
AD	0.003^**^	0.002^**^	0.034^*^	0.107	0.556	0.844	0.014^*^	0.093
RD	0.002^**^	0.001^**^	0.014^*^	0.281	0.664	0.568	0.010^*^	0.134

FA, fractional anisotropy; MD, mean diffusivity; AD, axial diffusivity; RD, radial diffusivity.*** *p* < 0.001, ** *p* < 0.01,* *p* < 0.05.

## 4 Discussion

In recent years, the incidence of cerebral small vessel disease (CSVD) has been continuously increasing, and common risk factors for CSVD include advanced age, hypertension, hyperlipidemia, etc. (20), which are also common risk factors for chronic heart failure (CHF) (21).

Clinical work and related studies have shown that CSVD has a common impact on different cognitive domains, mainly characterized by a decline in attention, memory, executive function and other domains than before (22). Cognitive function is also altered in patients with heart failure, and even subclinical changes in cardiac function are associated with neurodegeneration, cognitive dysfunction, and an increased incidence of clinical dementia (23). This study found that cognitive function was impaired in HF patients (the MoCA scale scores of these two groups were lower than the normal range), and which with impaired LVEF had a decrease in overall cognitive function compared to those with preserved LVEF, which was similarly observed in a recent cross-sectional study (24). It is generally believed that cognitive function impairment in CHF patients is mainly caused by cerebral blood perfusion insufficiency caused by the decrease of effective output. Pathologically, neurohormonal activation, oxidative stress, glial activation, inflammation, and loss of dendritic spines have all been proposed as contributors of cognitive dysfunction in heart failure (25).

In addition to overall cognitive function, our study also found that visuospatial/ executive functions, attention and delayed recall were positively correlated with LVEF in the HF patients.

The research on cognitive impairment in patients with HF suggest that HFpEF patients experience deficits in the same cognitive domains as HFrEF patients, especially in attention/processing speed, language/verbal fluency, delayed recall, executive function, etc., similar to the results previously published by Vogel (26). Compared with the results of this study, there was no significant language impairment in this study, which may be related to the following factors. The question setting for measuring language function in MoCA scale is relatively simple, and the majority of patients in this study had received cultural education at or above the secondary school, with good language abstraction and generalization abilities.

As factors both correlated with cognitive function, whether LVEF is related to CSVD and its different types has not been sufficiently explored in previous studies. The results of this study indicate that, among the four most common types of CSVD, CHF patients have a more severe incidence of white matter hyperintensities (WMH) (60.27%), which are independently associated with LVEF. Similar findings were reported earlier by Vogels et al. (27). The significant correlation between WMH and LVEF may be related to the mechanism of WMH. As mentioned in many reviews, hypoperfusion, dysfunction of the cerebral blood flow autoregulation, blood–brain barrier alterations, genetic factors, and inflammation are presumed to contribute to WMH (28, 29). Although some of these views are controversial, diffuse hypoperfusion has been suggested as its main mechanism (30). In addition to the cerebral diffuse hypoperfusion caused by chronic heart failure, we considered that thromboembolism caused by left ventricular (LV) systolic dysfunction is also the mechanism of this experimental result (8). In addition, when left ventricular pressure and volume are overloaded, the left atrial diameter (LAD) may increase. The enlargement of LAD in the pathogenesis of CSVD has also received attention. A previous study showed that increased LA volumes and reduced LA reservoir function correlated with WMH, suggesting the link between atrial dysfunction and CSVD (31), which may also be a potential mechanism for the correlation between WMH and CHF. From a pathophysiological perspective, ischemia on the capillary bed can lead to myelin loss and glial proliferation, which can be classically detected as WMH on T2 weighted sequences. A new technique, DTI, was used in our study to non-invasive quantify white matter integrity and provide *in vivo* evaluation of white matter microstructures in specific brain regions. Fractional anisotropy (FA) and mean diffusivity (MD) are the most commonly used parameters in voxel DTI modeling. Low FA or high MD parameters indicate a loss of water molecule directionality, typically caused by axonal degeneration and demyelination (32). Due to these abnormalities quantitatively predicting the development of lesions (WMH), they may be used to detect the early stages of CSVD. In our research, these imaging indicators were detected by TBSS method and used to explore the relationship between LVEF and CSVD.

Compared to CHF patients with LVEF≥50%, FA decreased significantly while MD, AD and RD increased significantly in CHF patients with LVEF <50% in our study. The FA value represents the degree of anisotropy of water molecule dispersion and measures the ability of water molecules to diffuse directionally in tissues, with a variation range of 0–1, and is an indicator of white matter integrity in the brain (33). Loss of myelin in white matter fibers, damage to axonal cell membrane integrity, and axonal transport speed all affect FA values (34). The decrease in FA value indicates that the brain white matter fiber structure of CHF patients with LVEF <50% has been damaged, resulting in a decrease in the anisotropy of water molecule dispersion. The MD value represents the overall diffusion size and is independent of the diffusion direction. The lower the MD value, the more limited the tissue structure (34). We observed a significant increase in MD in CHF patients with LVEF<50%, possibly due to an increase in free water molecules and expansion of the intercellular space leading to changes in the diffusion rate of water molecules. The AD value and RD value represent the degree of diffusion of water molecules parallel and perpendicular to the direction of fiber bundle movement, respectively. The increase in AD value is related to axonal damage, while the increase in RD value is related to demyelination (35, 36). Therefore, the above four diffusion index abnormalities indicate more significant changes in the microstructure of the brain white matter fiber bundles in the CHF patients with LVEF<50%, which are difficult to detect in traditional MRI sequences.

In our study, the brain regions with statistically significant changes in white matter fiber tracts included anterior thalamic radiation, corticospinal tract, cingulum (cingulate gyrus), cingulum (hippocampus), forceps major, forceps minor, inferior fronto-occipital fasciculus, inferior longitudinal fasciculus, superior longitudinal fasciculus, uncinate fasciculus and superior longitudinal fasciculus (temporal part). In previous studies, in cognitive impairment caused by CSVD, the damaged WM was mainly located in the prefrontal pathways of the thalamus and caudate lobe (e.g., anterior thalamic radiation, forceps minor, inferior fronto-occipital fasciculus, inferior longitudinal fasciculus, superior longitudinal fasciculus), which are significantly correlated with executive and attention functions (37). The forceps minor and forceps major are the main parts that make up the corpus callosum, which mainly transmit information in both hemispheres of the brain. The forceps minor connects the prefrontal cortex of both hemispheres and plays an important role in executive function (38); the forceps major connects the bilateral parietal cortex, and its integrity is related to visuospatial function and working memory (39). In the frontal lobe, fasciculus, which connects the orbital frontal cortex and plays an important role in attaching emotions to visuospatial function (40). Studies suggest that the human cingulum bundles are correlate with attention, memory, and executive function, including some of the most relevant cingulum connections, such as cingulate gyrus and hippocampus, which are even related to emotions (41). The projective fibers interconnect the cortical and subcortical structures, therefore in our study, a similar trend was also observed in the corticospinal tract. The above-mentioned lesions of the white matter fiber bundles involving the cortex, default network, limbic system, pyramidal tract, and other central nervous systems provide a pathophysiological basis for impaired cognitive function in CHF patients with decreased LVEF.

Furthermore, we discovered that lesions in these white matter fiber tracts were associated with impairments in visuospatial/executive function, attention, and delayed recall. This suggests that alterations in left heart function play a significant role in the development of cognitive impairment related to CSVD. Consequently, we conclude that the following pathological mechanisms may be involved: A decrease in left ventricular ejection fraction (LVEF) leads to the occurrence of CSVD, damaging various cortical–subcortical nerve circuits and resulting in cognitive impairment (30); When blood perfusion decreases, structural atrophy and damage occur in areas with poor blood supply, such as the frontal and temporal lobes, which affects cognitive function (42); Chronic cerebral hypoperfusion disrupts the integrity of the blood–brain barrier, allowing neurotoxic proteins to penetrate the vascular wall, leading to neuronal degeneration and apoptosis (28); Elderly individuals with congestive heart failure (CHF) have weak automatic regulation of cerebral blood flow and poor vascular compliance, and *β*-amyloid deposition is aggravated, leading to cognitive impairment when LVEF decreases (43).

This study is subject to several limitations. Firstly, as a cross-sectional observational study, it does not examine changes in white matter integrity throughout the progression of heart failure. The cross-sectional nature of the study design limits the ability to establish a temporal sequence among reduced left ventricular ejection fraction (LVEF), the onset of CSVD, and cognitive decline. Consequently, it is not possible to exclude the possibility of a reverse association, such as pre-existing occult CSVD leading to secondary cardiac dysfunction. This underscores the necessity for longitudinal studies to confirm the timing of these variables in future research.

Secondly, the study’s sample size is limited, particularly within the diffusion tensor imaging (DTI) subgroup. Only 37 participants (20 in the LVEF≥50% group and 17 in the LVEF<50% group) underwent DTI examinations, which may have contributed to the non-significant trends observed in diffusion metrics for certain white matter tracts. This small subgroup size may increase the risk of false-negative results and reduce the robustness of analyses for rare CSVD subtypes. Future research should aim to increase the sample size and include a healthy control group to facilitate a more comprehensive exploration.

Thirdly, cognitive function was evaluated exclusively using the Montreal Cognitive Assessment (MoCA) scale, which presents certain limitations in assessing specific cognitive domains. Notably, the MoCA scale exhibits low sensitivity in evaluating language function. Our data indicated no correlation between left ventricular ejection fraction (LVEF) and the language dimension of the MoCA (r = 0.024, *p* = 0.838), which may reflect the scale’s simplistic language-related items rather than an actual absence of language impairment in patients with chronic heart failure (CHF). The lack of a comprehensive neuropsychological battery may have obscured potential deficits in language fluency or working memory among CHF patients.

Fourthly, the study did not include an age- and gender-matched healthy control group, which constrains our ability to differentiate CHF-specific effects from those attributable to general aging processes. For instance, we are unable to ascertain whether the incidence of white matter hyperintensities (WMH) at 60.27% in CHF patients is significantly higher than that in age-matched healthy individuals, nor can we confirm whether cognitive impairment (MoCA scores <26 in both CHF groups) is unique to CHF or a common occurrence in the elderly population.

In conclusion, even after controlling for prevalent vascular risk factors, such as hypertension and diabetes, in our regression models, residual confounding factors may still be present. These factors could encompass depression, sleep quality, or variations in medication dosages, which may subtly affect cognitive scores. Improved data collection in future research will help mitigate the influence of these residual confounders.

## 5 Conclusion

Chronic heart failure (CHF) is significantly linked to the development of CSVD, particularly white matter hyperintensities (WMH, with an incidence of 60.27% among CHF patients), as well as to the severity of cognitive impairment. Notably, a reduced left ventricular ejection fraction (LVEF) is associated with poorer performance on the Montreal Cognitive Assessment (MoCA), with scores being significantly lower in the LVEF<50% group (21.79 ± 2.89) compared to the LVEF≥50% group (24.40 ± 2.23, *p* < 0.001). Furthermore, decreased LVEF is linked to more pronounced cerebral white matter microstructural damage, as indicated by lower fractional anisotropy (FA) and elevated mean diffusivity (MD), axial diffusivity (AD), and radial diffusivity (RD) in tract-based spatial statistics (TBSS) analysis (all *p* < 0.001). These findings suggest that LVEF may serve as a potential indicator associated with CSVD and cognitive decline in CHF patients. However, due to the cross-sectional design of this study, a causal relationship cannot be established, as it precludes determination of the temporal sequence between LVEF reduction, CSVD onset, and cognitive impairment.

## Data Availability

The original contributions presented in the study are included in the article/supplementary material, further inquiries can be directed to the corresponding authors.
